# Is complete myocardial revascularisation with total arterial coronary artery bypass graft a dream or reality?

**DOI:** 10.1002/hsr2.1532

**Published:** 2023-08-23

**Authors:** Aziz Momin, Redoy Ranjan, Venkatachalam Chandrasekaran

**Affiliations:** ^1^ Department of Cardiac Surgery St George's University Hospitals NHS Foundation Trust London UK; ^2^ Department of Cardiac Surgery Bangabandhu Sheikh Mujib Medical University Dhaka Bangladesh

**Keywords:** CABG, coronary artery bypass graft surgery, long‐term, mortality, surgical myocardial revascularization, survival, total arterial CABG

## 1 INTRODUCTION

In 1986, Loop et al. first illustrated the long‐term prognostic benefits of left internal mammary artery (LIMA) to left anterior descending graft.^1^ Surgical myocardial revascularisation with coronary artery bypass graft (CABG) surgery is the most commonly performed and preferred strategy for multivessel coronary artery disease.^1^, ^2^ In the absence of large enough powered randomized controlled trials (RCTs), recent studies observed that total arterial revascularisation (TAR) utilizing bilateral internal mammary artery (IMA) and radial conduits carry better longevity and reduces postoperative morbidity, particularly in early graft failure, recurrent angina, and redo‐CABG surgery.^3^, ^4^ However, the potential challenge of TAR‐CABG surgery, especially among left main coronary artery disease, depends on the premise that TAR will have a better graft patency rate and postoperative health‐related quality of life.^4^, ^5^, ^6^ Here, we describe the long‐term (≥6 months) survival benefits of myocardial revascularisation with multiple arterial CABG surgery over 20 years in the United Kingdom.

## 2 METHODS

A total of 2979 consecutive isolated elective CABG patients at St Georges University Hospital NHS Foundation Trust from April 1999 to March 2020 were studied, and the last day of the census was May 5, 2021. The study population was distributed in four groups—bilateral internal mammary artery + radial (BIMA+R; *n* = 431), single internal mammary artery + radial ± vein (SIMA+R±V; *n* = 823), single internal mammary artery − radial ± vein (SIMA−R±V; *n* = 823), and radial ± vein (R±V; *n* = 160) groups. The institutional review board clearance was waived as this retrospective analysis of prospectively collected data under the adult National Institute for Cardiovascular Outcomes Research UK database. Study inclusion criteria were isolated CABG with or without prior history of heart surgery, and patients with concomitant valvular, congenital heart diseases were excluded from the study. Multiple arterial graft CABG populations (BIMA+R and SIMA+R±V) have ≥3 arterial grafts, including sequential arterial grafts with the LIMA, right internal mammary artery (RIMA), and radial artery with or without venous grafts. A statistical package for the social sciences 25.0 version software was utilized to analyze the data, and a *p* value ≤ 0.05 is considered statistically significant.

## 3 RESULTS

**Figure 1:**
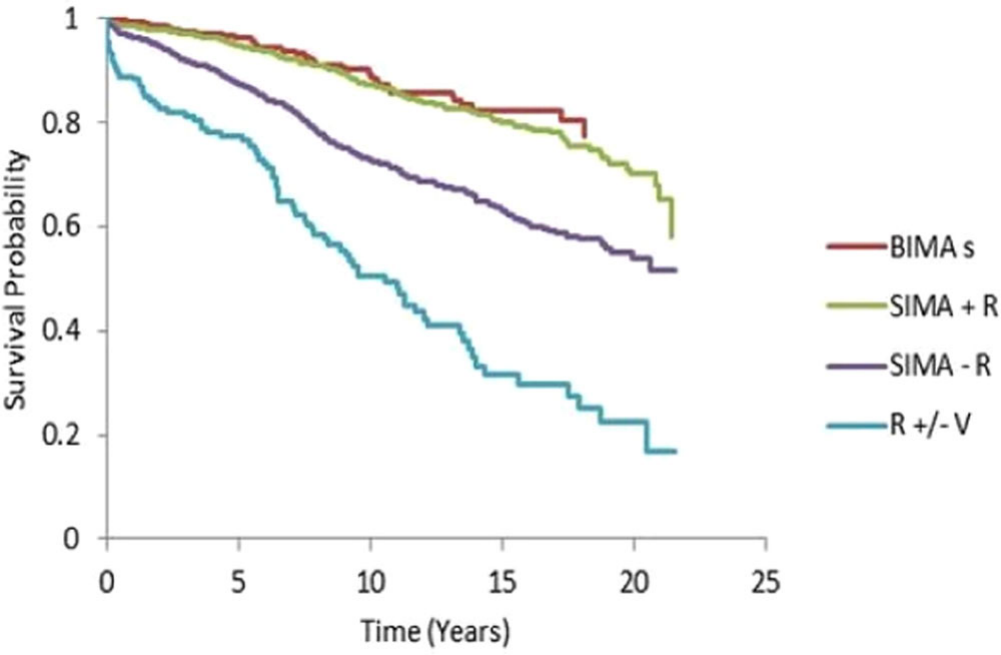
A K–M survival curve for IMAs, radial artery, and vein subgroups. Hazard ratio with 95% confidence interval was utilized to evaluate the survival rate of four study groups. BIMA+R, bilateral internal mammary artery + radial; K–M, Kaplan–Meier; IMA, internal mammary artery; R±V, radial ± vein; SIMA+R, single internal mammary artery + radial; SIMA−R, single internal mammary artery ‐ radial.

We found that males (~80%) are predominant, and the median age was 61 years (interquartile range [IQR]: 55–68), 63 years (IQR: 57–69), 72 years (IQR: 65–77), and 71 years (IQR: 65–77) in BIMA+R, SIMA+R±V, SIMA−R±V, and R±V groups, respectively. Gender distribution, male versus female, was 90.7% versus 9.3%; 81.2% versus 18.8%; 78.5% versus 21.5%; and 76.3% versus 23.7% among the BIMA+R, SIMA+R±V, SIMA−R±V, and R±V groups, respectively. Multiple arterial CABG (≥3 grafts) was performed in 45.5% and 39.9% cases among BIMA+R and SIMA+R±V populations, respectively. Further, 35.3% and 34.4% of patients had multiple (≥3) mixed arterio‐venous grafting in SIMA−R±V and R±V CABG groups. We found that overall survival times were 19.1, 18.6, 15.8, and 10.9 years with BIMA+R, SIMA+R±V, SIMA−R±V, and R±V groups, respectively. Redo CABG was performed in four cases; two cases in each SIMA+R±V (0.2%) and SIMA−R±V (0.1%) group. A statistically significant (*p* ≤ 0.05) long‐term (≥6 months) survival advantage for multiple arterial grafting was demonstrated, especially TAR, over all other combinations except single internal mammary artery + radial artery grafting (Figure 1).

## 4 DISCUSSION

This study observed multiple arterial graft CABG population had better long‐term survival, and the poorest outcome was in the R±V group with no IMA graft, similar to recently published articles where saphenous vein grafts are more prone to developing early graft failure and develop recurrent angina attacks, leading to poor quality of life and increased reintervention rate.^6^, ^7^, ^8^ We found the mean survival age was similar (19.1 vs. 18.6 years) among BIMA+R and SIMA+R±V groups might be due to the age at CABG surgery being identical and both belonging to multiple arterial graft CABG populations. In an RCT, Gaudino et al. observed that radial‐artery grafts have a higher graft patency rate and a low adverse cardiac event over 5 years of follow‐up, similar to our study results.^9^

The radial artery is believed to be disease‐free with a good caliber and length, relatively resistant to the atherosclerosis process and has a good muscle layer facilitating better graft patency.^7^, ^8^, ^9^ In an international study coordinated in the United Kingdom, a randomized controlled trial pioneered by Taggart et al.^10^ evaluated the long‐term mortality rate of bilateral versus single IMA grafts for CABG and observed no significant difference in all‐cause of mortality over 10 years of follow‐up, which is similar to other published articles.^5^, ^6^, ^7^, ^8^, ^9^ Moreover, Royse et al.,^11^ Rocha et al.,^12^ and Rayol et al.,^13^ observed better long‐term survival benefits of multiple arterial CABG populations and encouraged the utilization of more arterial conduits, identical to the current study results.

The preservation of graft patency is influenced by vascular endothelial nitric oxide (NO) and increased stress within the arterial circulation.^14^, ^15^ Nitric oxide helps maintain vascular tone, preventing platelet aggregation, white blood cell activation, thrombus formation, and smooth muscle cell proliferation. However, arterial conduits, particularly the radial artery, exhibit superior endothelium‐dependent relaxation and remodeling under increased stress. In contrast, vein grafts show a decrease in the biological effects of NO and changes in gene expression, leading to vascular smooth muscle cell proliferation, acceleration of degenerative process, and atherosclerosis, which results in a poor graft patency rate.^14^, ^15^

According to the existing literature,^8^, ^10^, ^13^, ^16^ our revascularisation strategy was to achieve complete myocardial revascularisation utilizing more arterial conduits and arterial grafts (with sequential arterial grafts if needed) based on a distinct preoperative plan on angiographic findings to accomplish total arterial CABG. Further, existing articles^6^, ^7^, ^8^ found BIMA harvesting poses challenges for sternal wound infection; we found no significant long‐term adverse outcome associated with utilizing BIMA grafts over 20 years. Insofar as we know, this is the most extensive TAR‐CABG study in the United Kingdom; however, its nonrandomized retrospective observational methods put some methodological limitations despite enough statistical power. Although the study sample was male predominant and based on a heterogeneous group from a single institute, the survival rate in each study group and the odds ratio of potential risk factors are topics of high interest. Nevertheless, our redo‐CABG cases were performed due to a new coronary lesion having study limitations as specific data on‐site, territory, and percentage of the lesion is lacking. Furthermore, we believe an RCT or large observational study based on angiographic evaluation of graft patency rate following multi‐arterial CABG will shed more light on this study outcome.

## 5 CONCLUSION

Multiple arterial CABG surgery is feasible and has excellent long‐term survival benefits compared to the mixed arterio‐venous graft population over 20 years of follow‐up.

## AUTHOR CONTRIBUTIONS

**Aziz Momin**: Conceptualization; formal analysis; methodology; resources; supervision; validation; visualization; writing—review and editing. **Redoy Ranjan**: Conceptualization; formal analysis; methodology; resources; validation; visualization; writing—original draft; writing—review and editing. **Venkatachalam Chandrasekaran**: Conceptualization; methodology; resources; supervision; validation; writing—review and editing.

## CONFLICT OF INTEREST STATEMENT

Redoy Ranjan is an Editorial Board member of Health Science Reports, and a coauthor of this article. To minimize bias, they were excluded from all editorial decision‐making related to the acceptance of this article for publication.

## ETHICS STATEMENT

In accordance with the National Research Ethics Service, this retrospective study, using data already collated as patients received their usual care, did not require research ethics committee approval but adhered to international standards for GDPR (General Data Protection Regulation).

## TRANSPARENCY STATEMENT

The lead author Redoy Ranjan affirms that this manuscript is an honest, accurate, and transparent account of the study being reported; that no important aspects of the study have been omitted; and that any discrepancies from the study as planned (and, if relevant, registered) have been explained.

## Data Availability

The research data used to support the findings of this study are available from the corresponding author of this study upon request.

## References

[1] Loop FD , Lytle BW , Cosgrove DM , et al. Influence of the internal‐mammary‐artery graft on 10‐year survival and other cardiac events. N Engl J Med. 1986;314:1‐6. 3484393 10.1056/NEJM198601023140101

[2] Marasco S . Total arterial revascularization. Oper Tech Thorac Cardiovasc Surg. 2016;21:20‐30.

[3] Dewantoro D , Nenna A , Satriano U , Chello M , Spadaccio C . Advantages and disadvantages of total arterial coronary artery bypass graft as compared to venous coronary artery bypass graft. Vessel Plus. 2018;2:20.

[4] Suzuki T , Asai T , Nota H , Kinoshita T , Fujino S . Impact of total arterial reconstruction on long‐term mortality and morbidity: off‐pump total arterial reconstruction versus non‐total arterial reconstruction. Ann Thorac Surg. 2015;100:2244‐2249. 26234659 10.1016/j.athoracsur.2015.05.034

[5] Tranbaugh RF , Dimitrova KR , Lucido DJ , et al. The second‐best arterial graft: a propensity analysis of the radial artery versus the free right internal thoracic artery to bypass the circumflex coronary artery. J Thorac Cardiovasc Surg. 2014;147:133‐140. 24100104 10.1016/j.jtcvs.2013.08.040

[6] Yanagawa B , Verma S , Mazine A , et al. Impact of total arterial revascularization on long term survival: a systematic review and meta‐analysis of 130,305 patients. Int J Cardiol. 2017;233:29‐36. 28185702 10.1016/j.ijcard.2017.02.010

[7] Dimitrova KR , Hoffman DM , Geller CM , Dincheva G , Ko W , Tranbaugh RF . Arterial grafts protect the native coronary vessels from atherosclerotic disease progression. Ann Thorac Surg. 2012;94:475‐481. 22727250 10.1016/j.athoracsur.2012.04.035

[8] Aldea GS , Bakaeen FG , Pal J , et al. The Society of Thoracic Surgeons clinical practice guidelines on arterial conduits for coronary artery bypass grafting. Ann Thorac Surg. 2016;101:801‐809. 26680310 10.1016/j.athoracsur.2015.09.100

[9] Gaudino M , Benedetto U , Fremes S , et al. Radial‐artery or saphenous‐vein grafts in coronary‐artery bypass surgery. N Engl J Med. 2018;378:2069‐2077. 29708851 10.1056/NEJMoa1716026

[10] Taggart DP , Benedetto U , Gerry S , et al. Bilateral versus single internal‐thoracic‐artery grafts at 10 years. N Engl J Med. 2019;380:437‐446. 30699314 10.1056/NEJMoa1808783

[11] Royse A , Pawanis Z , Canty D , et al. The effect on survival from the use of a saphenous vein graft during coronary bypass surgery: a large cohort study. Eur J Cardiothorac Surg. 2018;54:1093‐1100. 29893823 10.1093/ejcts/ezy213

[12] Rocha RV , Tam DY , Karkhanis R , et al. Long‐term outcomes associated with total arterial revascularization vs non–total arterial revascularization. JAMA Cardiology. 2020;5:507‐514. 32074240 10.1001/jamacardio.2019.6104 PMC7042852

[13] Rayol SC , Eynde JV , Cavalcanti LRP , et al. Total arterial coronary bypass graft surgery is associated with better long‐term survival in patients with multivessel coronary artery disease: a systematic review with meta‐analysis. Braz J Cardiovasc Surg. 2021;36:78‐85. 33594864 10.21470/1678-9741-2020-0653 PMC7918394

[14] Lüscher TF , Diederich D , Siebenmann R , et al. Difference between endothelium‐dependent relaxation in arterial and in venous coronary bypass grafts. N Engl J Med. 1988;319:462‐467. 3136329 10.1056/NEJM198808253190802

[15] Otsuka F , Yahagi K , Sakakura K , Virmani R . Why is the mammary artery so special and what protects it from atherosclerosis? Ann Cardiothorac Surg. 2013;2:519‐526. 23977631 10.3978/j.issn.2225-319X.2013.07.06 PMC3741888

[16] Torregrossa G , Amabile A , Williams EE , Fonceva A , Hosseinian L , Balkhy HH . Multi‐arterial and total‐arterial coronary revascularization: past, present, and future perspective. J Card Surg. 2020;35:1072‐1081. 32293059 10.1111/jocs.14537

